# Correlation of the Imbalance in the Circulating Lymphocyte Subsets With C-Reactive Protein and Cardio-Metabolic Conditions in Patients With COVID-19

**DOI:** 10.3389/fimmu.2022.856883

**Published:** 2022-05-06

**Authors:** Anton V. Tyurin, Milyausha K. Salimgareeva, Ildar R. Miniakhmetov, Rita I. Khusainova, Alexandr Samorodov, Valentin N. Pavlov, Julia Kzhyshkowska

**Affiliations:** ^1^ Internal Medicine Department, Bashkir State Medical University, Ufa, Russia; ^2^ Republic Medical Genetic Center, Ufa, Russia; ^3^ Medical Genetics Department, Bashkir State Medical University, Ufa, Russia; ^4^ Department of Pharmacology, Bashkir State Medical University, Ufa, Russia; ^5^ Urology Department, Bashkir State Medical University, Ufa, Russia; ^6^ Institute of Transfusion Medicine and Immunology, Medical Faculty Mannheim, Heidelberg University, Mannheim, Germany; ^7^ German Red Cross Blood Service Baden-Württemberg – Hessen, Mannheim, Germany; ^8^ Laboratory for Translational Cell and Molecular Biomedicine, Tomsk State University, Tomsk, Russia

**Keywords:** COVID-19, C-reactive protein, lymphocytes, metabolic syndrome, cardiovascular pathology, systemic inflammation

## Abstract

The immune system is severely compromised in patients with COVID-19. The representative group of 43 patients were selected from the cohort of 342 patients with COVID-19 and pneumonia. This group of 43 patients was examined for the levels of C-reactive protein, biomarker of systemic inflammation, and for the subsets of adaptive immune cells. The immunological parameters were correlated with the metabolic parameters and cardiovascular pathology history. We identified that a decrease in the absolute number of T-lymphocytes, T-cytotoxic, T-activated and B-lymphocytes correlated with the higher levels of CRP. The absolute number of T-helpers and the absolute number of double positive T-lymphocytes positively correlated with the levels of iron in serum (Z= 0,310 and Z=0,394). The absolute numbers of T-activated lymphocytes positively correlated with serum levels of LDH (Z = 0,422), ferritin (Z = 0,407) and iron (Z = 0,418). When studying subpopulations of lymphocytes, depending on the combined pathology, we found that the absolute numbers of B-lymphocytes and double positive T-lymphocytes in the peripheral blood were significantly reduced in patients with arterial hypertension (p=0,0074 and p=0,0227, correspondingly). The increased levels of NK cell were found in patients with a history of coronary heart disease (p=0,0108). In addition, we found that deficiencies in the adaptive immune system correlated with the deficiencies in iron metabolism. The cardiovascular pathology upsets the balance in the adaptive and innate immune system in the circulation of patient with severe COVID-19.

## Introduction

Coronaviruses are a large group of RNA-containing viruses that cause both colds with mild clinical course and severe forms, accompanied by damage to the lower respiratory tract with respiratory failure. A new variant of the virus, named SARS-Cov-2, was first identified in China in 2019 and caused a pandemic that has not yet been localized. To date, more than 176 million confirmed cases have been registered worldwide, including 5,15 million in Russia, and it`s amount continues to grow (1). The main clinical manifestations of coronavirus infection include fever, shortness of breath, dry cough, muscle aches, sweating, diarrhea, and pulmonary disease such as interstitial pneumonia. In the severe cases, the process affects not only the lungs, but also other internal organs, and it leads to the development of multiple organ failure and death (2).

To date, there are no absolutely effective pharmacotherapy regimens, which makes it necessary to further study the nature of the disease, its pathogenesis and developmental mechanisms in order to search for potential targets of therapeutic action. Studies demonstrate that there is a decrease in the absolute number of lymphocytes in patients with COVID-19, including CD4 + and CD8 + T-lymphocytes (3), while the antiviral activity of the immune system depends on the activation of CD8 T-cells, the increase of its number is necessary for the lysis of infected cells and the fastest recovery (4). The factors affecting the development of lymphopenia are not completely understood, most observations indicate a correlation between the severity of the infection and the severity of lymphopenia. One of the quantitative characteristics of the inflammatory process and a predictor of the disease severity is C-reactive protein (CRP) (5). Studies on the effect of CRP levels on the development of lymphopenia in general and on the effect on lymphocyte subpopulations are fragmentary. In the study by Acar et.al, the ratio of the number of lymphocytes in peripheral blood to the level of C-reactive protein was higher in patients who died from COVID-19 compared with survivors (0,03 and 0,36, p <0,05) (6). In another study, correlations were found between the levels of C-reactive protein and lymphocyte subpopulations, for CD3 and CD8 they reached statistical significance (p = 0,01 and p = 0,004, respectively) (7).

Therefore, the purpose of this study was to determine the characteristics of peripheral blood lymphocyte subpopulations and to search for associations with clinical and laboratory parameters of the COVID-19 disease.

## Material and Methods

### Patients

The study included 342 patients with COVID-19, complicated by pneumonia, hospitalized at the clinic of the Bashkir State Medical University. All patients received treatment according to the Temporary National Recommendations and local protocols. Monitoring of somatic status, laboratory parameters and CT scan of lungs were carried out. Out of the total patients’ cohort, 43 patients were selected by blind randomization to ensure representative study group. Blood samples for the isolation of peripheral blood lymphocytes were obtained at the moment of hospitalization before any treatment onset started. The peripheral blood lymphocytes of the 43 patients study group were analyzed by immunophenotyping (IPT). The main characteristics of the total and study groups are presented in **Table 1**.

**Table 1:** Basic characterisation of total COVID-19 patients’ cohort and cohort selected for the lymphocyte analysis.

Parameter	Normal value	Total sample N= 342; Me (Q1-Q3)	Immunophenotyped sample N= 43; Me (Q1-Q3)
Age, years		59,00 (48,0- 67,0)	60,0 (50,0-69,0)
Hospitalization, days		12,00 (10,0-14,0)	9,0 (8,0-12,0)
Before hospitalization, days		7,00 (5,0-9,0)	7,0 (5,0-10,0)
Leukocytes, 1 * 10^6^ / l	4,0-9,0	5,10 (3,7-7,0)	4,84 (4,02-6,75)
Platelets, 1* 10^3^ / l	150-400	195,0 (157,0-252,0)	180,0 (143,0-229,0)
Erythrocytes, 1 * 10^12^ / l	3,5-5,5	4,6 (4,26-4,88)	4,48 (4,18-4,72)
Neutrophils, %	40-70	71,4 (61,2-78,4)	62,5 (54,8-73,5)
Lymphocytes, %	19-45	23,2 (15,7-31,7)	24,0 (19,5-34,3)
Neutrophils / Lymphocytes ratio	2,47±0,65	3,08 (1,89-4,84)	2,58 (1,64-3,68)
CRP, mg / l	0-9	44,9 (24,4-73,7)	29,6 (18,0-60,0)
Creatinine, μmol / l	44-124	91,7 (83,0-104,0)	95,6 (80,2-105,5)
AST, Unit	2-35	29,4 (22,3-42,2)	25,6 (20,4-34,8)
ALT, Unit	4-41	26,5 (18,6-43,3)	26,5 (18,1-33,8)
KFK, Ed	2-171	122,5 (70,0-234,0)	111,0 (73,0-163,0)
LDH, Unit	5-480	360,0 (301,0-449,0)	323,0 (264,0-430,0)
Iron, μmol / l	6,6 - 26	8,0 (5,2-12,2)	11,0 (6,1-17,0)
Ferritin, mcg / l	20 - 250	380,2 (188,1-631,4)	287,0 (156,05-540,5)

AST, aspartate aminotransferase; ALT, alanine aminotransferase; KFK, creatine phosphokinase; LDH, lactate dehydrogenase.

### Analysis of Clinical Parameters

The hematological parameters were analyzed using an automatic analyzer CELL-DYN Sapphire (Abbot, USA), the biochemical parameters were studied using an automatic analyzer CA-800 (Furuno Electric Co. Ltd., Japan). Computed tomography was performed using an Optima CT660 device (General Electric, USA). COVID-19 diagnosis was done by real-time polymerase chain reaction (RT-PCR) (Vector-Best COVID-19 RT-qPCR kit, Russia) of viral nucleic acids form throat swab samples.

### C-Reactive Protein Analysis

Samples were obtained from peripheral blood in EDTA containing tubes and processed accordingly (1,500 g, 15 min). Plasma high sensitive CRP levels were determined using Wide Range C-Reactive Protein (wr-CRP) Turbidimetric Immunoassay Kit (Toronto BioScience, USA) following manufacturer instructions.

### Metabolic Syndrome Analysis

Diagnostics of the metabolic syndrome included the assessment of body mass index and the detection of arterial hypertension, hypercholesterolemia, diabetes mellitus, and pathology of the coronary and cerebrovascular arteries according to the medical records of patients in the Unified State Information System in Healthcare (Russia). Diagnosis was available before COVID-19 disease. Body mass index was calculated using the formula kg/m^2^ where kg is a person’s weight in kilograms and m^2^ is their height in meters squared.

### Lymphocyte Subpopulations Analysis

10 ml from peripheral venous blood were used. Sample preparation for cytometric counting was performed according to the recommendations given in the standardized technology (8). Phenotype of blood lymphocytes was analyzed by flow cytometry (Beckman Coulter, USA). Combinations of fluorescently labeled of antibodies were designed according to the principles of forming panels for multicolor cytometric studies described in the literature (9, 10). Following antibodies were used: tetraCHROME CD45-FITC/CD56-PE/CD19-ECD/CD3-PC5 Antibody Cocktail (Beckman Coulter, cat.N 6607073); CD3-FITC/HLA-DR-PE Antibody Cocktail (Beckman Coulter, cat.N A07737), CD45-Pacific Blue (Beckman Coulter cat.N А74763); Flow-Count (Beckman Coulter cat.N 7547053).

Erythrocytes were removed from the samples using a no-wash technology with VersaLyse Lysing Solution (Beckman Coulter, USA). Absolute values were obtained in a single platform system using FlowCount ™ reagent (Beckman Coulter, USA). Samples were analyzed on a Navios ™ flow cytometer (Beckman Coulter, USA) equipped with three diode lasers 405, 488, 638 nm and 10 fluorescence detection channels. In each sample, at least 70,000 lymphocytes were analyzed. Cytofluorometric data were processed using Navios Software v.1.2 (Beckman Coulter, USA). Study contained 9 subpopulations - lymphocytes (CD45 bright), B-cells (CD3-CD19+), T-cells (CD3+CD19-), T-helpers (CD3+CD4+CD8-), T-cytotoxic (CD3+CD8+CD4-), T-NK cells (CD3+CD56+), double positive T-lymphocytes (CD4+CD8+), True natural killer cells (CD3-CD56+), activated T cells (CD3+HLA-DR+).

### Statistical Methods

We used Statistica 13.0 software (TIBCO Software, Palo Alto, CA, USA) to all comparison, the Shapiro-Wilk test to test the normal distribution of continuous variables, the Student’s t-test and the Mann-Whitney test with Wilcoxon’s posterior signed rank test to compare the groups and Spearman teas to estimate correlations. Results were expressed as mean with SD for normal distribution, otherwise the median with interquartile range (Q1 - Q3). Differences were considered statistically significant at p <0,05.

## Results

### Clinical Characterization of COVID-19 Patients

The basic clinical and biochemical parameters analysed in blood of patients in the total COVID-19 cohort and COVID-19 patients examined of the analysis of lymphocyte subpopulation and levels of C-reactive protein did not differ, indicating that selected group of 43 patients is representative for the total COVID-19 cohort (**Table 1**). All patients had bilateral polysegmental pneumonia on the chest CT scans. In the total cohort, CT-1 (up to 25% of the lesion) was detected in 42 patients, CT-2 (25% -50% of the lesion) in 148, CT-3 (51% -75% of the lesion) in 128 and CT-4 (more 76% lesion) in 15 patients. In the study group, CT-1 was found in 8 patients, CT-2 in 26, CT-3 in 9 patients. Patients included in our study had moderate disease level according to the WHO Clinical Progression Scale [www.who.int]. Patients, included in our study, did not require and were not subjected to non-invasive ventilation or high-flow oxygen. 28 patients had no oxygen supply (score 4), 15 patients received oxygen by face mask (score 5). No statistically significant differences have been identified in the circulating CRP levels in the total cohort and in the study group. In 17 patients CRP levels were within the reference (0-9 mg/l) values (Group 1). The remaining patients we divided into 2 subgroups: with CRP levels of 10-50 mg/l (Group 2) and with CRP levels over 51 mg/l (Group 3). For all three groups, the main clinical and laboratory characteristics are summarized in the **Table 2**.

**Table 2:** Clinical and laboratory characteristics of COVID-19 patients divided into 3 groups according to the CRP levels.

Parameter	Normal value	C-RP <10mg/ lGr. 1 N=17Me (Q1 -Q3)	C-RP 10-50mg/l Gr. 2 N=16Me (Q1 -Q3)	C-RP >51mg/l Gr. 3 N=10Me (Q1 -Q3)
Age, years		58,0 (44,0-69,0)	58,50 (49,5-62,5)	63,0 (60,0-70,0)
Diabetes		1 (5,8%)	3 (18,7%)	0
Hypercholesterolemia		3 (17,6%)	0	0
Arterial hypertension		8 (47,0 %)	7 (43,7%)	7 (70,0%)
Coronary artery disease		3 (17,6%)	2 (12,5%)	2 (20,0%)
Cerebrovascular disease		7 (41,1%)	2 (12,5%)	4 (40,0%)
Hospitalization, days		8,0 (8,0-9,0)	9,50 (8,0-11,0)***	13,0 (10,0-14,0)**
Before hospitalization, days		8,0 (6,0-10,0)	5,50 (4,0-8,5)	5,5 (4,0-12,0)
BMI, kg/m^2^, M±SD	18,5-24,99	28,45±5,65	28,43±3,16	30,07±5,71
Leukocytes, 1 * 10^6^ / l	4,0-9,0	4,84 (4,5-6,11)	4,99 (4,1-5,95)	5,14 (3,66-7,85)
Platelets, 1* 10^3^ / l	150-400	215,0 (180,0-257,0)*	171,00 (142,5-209,5)	152,5 (98,0-188,0)**
Erythrocytes, 1 * 10^12^ / l	3,5-5,5	4,3 (4,07-4,87)	4,66 (4,21-4,69)	4,4 (4,19-4,62)
Neutrophils, %	40-70	57,8 (51,5-62,0)	64,80 (53,9-74,65)	73,0 (63,1-74,6)**
Lymphocytes, %	19-45	32,5 (29,4-35,8)*	21,80 (18,95-33,15)	20,25 (19,1-23,3)**
N / L ratio	2,47±0,65	1,81 (1,43-2,34)	2,86 (1,75-3,96)	3,543 (2,68-3,9)**
Lymph. / CRP ratio	–	6,5 (5,8-7,16)*	1,23 (0,84-2,12)***	0,309 (0,22-0,40)**
CRP, mg / l	0-9	5,2 (2,9-7,31)*	18,25 (12,0-26,31)***	67,54 (57,7-78,0)**
Creatinine, μmol / l	44-124	87,0 (76,5-101,1)	96,20 (81,2-104,75)	104,7 (95,6-123,8)
AST, Unit	2-35	26,8 (20,4-34,8)	24,20 (19,45-33,75)	27,4 (24,1-45,1)
ALT, Unit	4-41	27,0 (17,2-28,1)	29,50 (19,75-44,45)	24,3 (19,5-28,2)
KFK, Ed	2-171	106,0 (67,0-137,0)	107,50 (74,0-170,5)	142,0 (122,0-163,0)
LDH, Unit	5-480	295,0 (251,0-340,0)	311,50 (260,5-416,0)	435,0 (354,0-561,0)**
Iron, μmol / l	6,6 - 26	12,0 (11,2-18,6)	10,95 (7,25-17,8)***	6,0 (5,8-6,6)**
Ferritin, mcg / l	20 - 250	152,0 (65,3-238,0)*	407,55 (293,8-671,9)	541,2 (403,7-564,3)**

* level p <0.05 Gr. 1 vs Gr. 2; ** level p <0.05 Gr. 1 vs Gr. 3; *** level p <0.05 Gr. 2 vs Gr. 3.

The average age of patients in the groups 1 and 2 was similar (58 vs. 58,5 years). In group 3 the average age was 63 years, however, the difference between group 1 and 2 were not statistically significant (**Table 2**). The duration of hospitalization was statistically significantly higher in patients with a higher CRP level (8 vs. 13 days, p=0,0032 for group 1 and 3 and 9,5 vs. 13, p=0,0048 for group 2 and 3) arguing towards the negative effect of systemic inflammation for the COVID-19 disease severity. The increase in CRP levels correlated with the decrease in the lymphocytes amounts (Z= -0,467). CRP levels correlated also with the percentage of neutrophils (Z= 0,459) and with the decrease in platelets (Z= -0,440). No correlations have been identified for CRP levels with the total number of leukocytes and erythrocytes.

The analysis of biochemical parameters revealed a statistically significant increase in the level of lactate dehydrogenase (LDH) in the third group with CRP levels over 51 mg/l compared to the first group (295 vs. 495, p=0,002). CRP groups 2 and 3 had increased levels of ferritin with statistical significance for group 3 (295,0 vs. 435,0, p=0,0054), and a decrease in the level of serum iron, most pronounced in group 3 (12,0 vs. 6,0, p=0,014). There was an increase in the levels of creatine phosphokinase in third group, which, however, did not reach statistical significance. The levels of aspartate aminotransferase and alanine aminotransferase did not change in the study groups compared to reference values, which indicates the absence of a direct cytolytic process in the liver at this stage of the disease.

Remarkable is the ratio of the number of lymphocytes to the level of CRP, which statistically significantly differs between CRP groups 2 and 3 (6,5 vs. 1,23 and 6,5 vs. 0,309, with p=0,0032 and p=0,0012, correspondingly). Such ratio was previously used to assess the prognostic parameter in patients with lung or colon cancer (11, 12). Our data suggest that the lymphocyte/CRP ratio can be used in clinical practice as a sensitive parameter for assessing the inflammatory status in patients also in the disorders of infectious etiology.

### Metabolic Syndrome and C-Reactive Proteins Levels in COVID-19 Patients

COVID-19 patients were analyzed for the clinical manifestations of metabolic syndrome: high body mass index (BMI), and for the presence associated clinical conditions - diabetes mellitus, hypercholesterolemia, hypertension, ischemic heart disease, cerebrovascular pathology (**Table 2**). Notable that in all studied groups, BMI exceeded normal values and ranged from overweight in the groups 1 (28,45) and 2 (28,43) to the obesity I in the group 3 (30,07). We did not find statistically significant correlations between CRP levels with the incidence of following clinical conditions: diabetes, hypercholesterolemia, coronary artery disease, cerebrovascular disease. The incidence of arterial hypertension was 47% in the group 1; 43,7% in the group 2, and 70% in the group 3, but the differences did not reach the statistical significance due to high variability in the patients. When assessing the CRP level depending on the presence of AH, in the group with high blood pressure, the average CRP levels were higher (Me = 18,8 [2,0-23,8] and Me = 18,8 [1,8 -60,9], respectively), the differences did not reach the level of statistical significance, most likely due to big individual variations.

### Analysis of Lymphocyte Subpopulations in 3 Groups Defined by C-Reactive Protein

**Figure 1:**
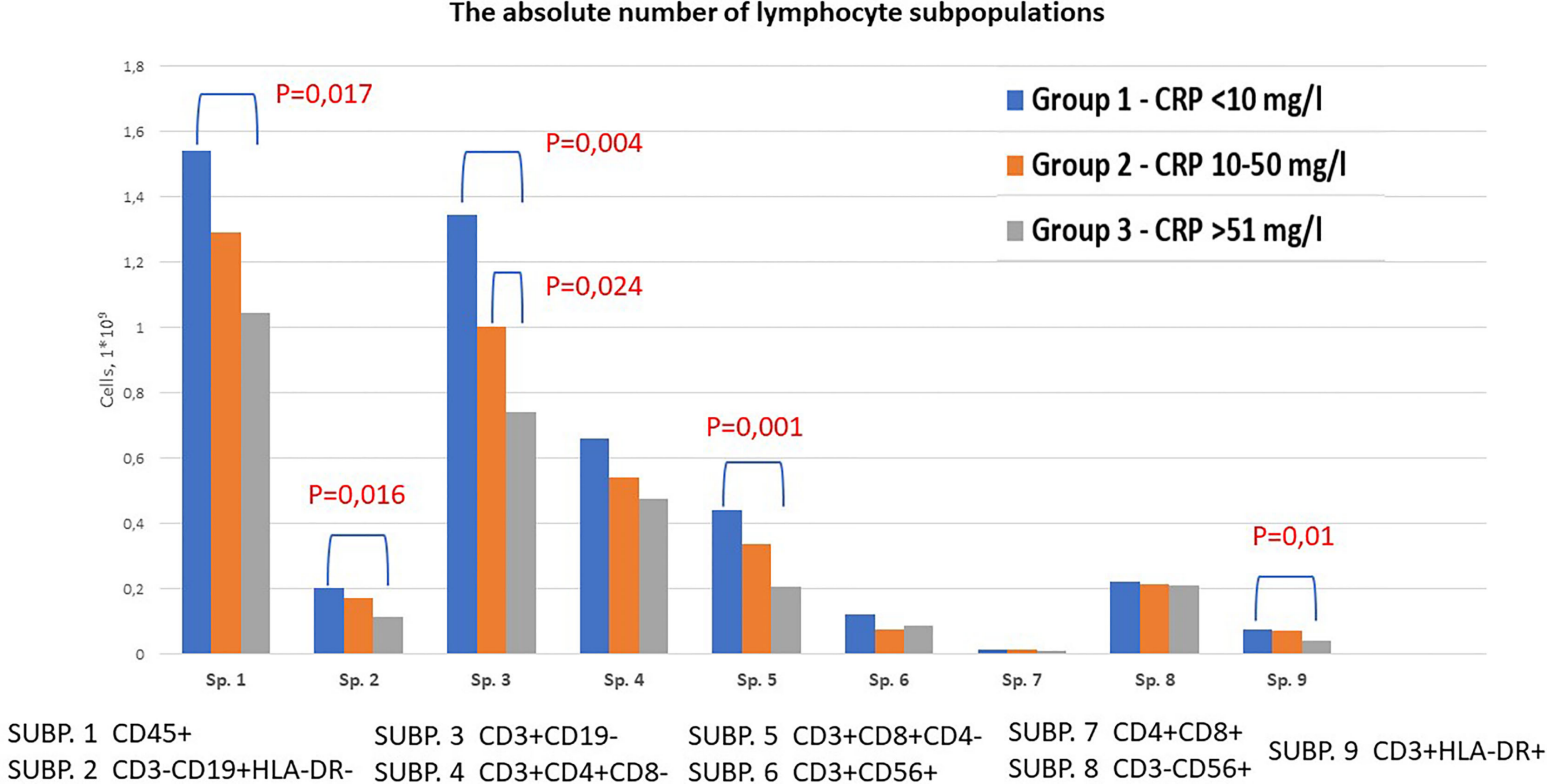
Content of lymphocyte subpopulations depending on CRP levels in patients with COVID-19. Venous blood out of 43 patients was analyzed using flow cytometry to identify SUBP1 – total lymphocytes (CD45 bright), SUBP2 - B-cells (CD3-CD19+), SUBP3 - T-cells (CD3+CD19-), SUBP4 - T-helpers (CD3+CD4+CD8-), SUBP5 - T-cytotoxic (CD3+CD8+CD4-), SUBP6 - T-NK cells (CD3+CD56+), SUBP7 - double positive T-lymphocytes (CD4+CD8+), SUBP8 - True natural killer cells (CD3-CD56+), SUBP9 - activated T cells (CD3+HLA-DR+). COVID-19 patients were divided in 3 groups according to the CRP levels: group 1 <10 mg/l; group 2 10-50 mg/l; group 3 >50 mg/l. Statistical analysis was carried out using Mann-Whitney test.

We investigated the absolute and relative number of the main subpopulations of lymphocytes in groups with different concentrations of CRP (**Figure 1** and **Supplementary Tables 1, 2**). We found decreased total absolute number of lymphocytes (1,043 vs. 1,541; p=0,032), B-cells (0,112 vs. 0,202; p=0,027), T-cells (0,742 vs. 1,345; p=0,012), T-cytotoxic (0,206 vs. 0,440; p=0,018) and T-activated cells (0,041 vs. 0,076; p=0,021) in group 3 compared to group 1. Also, the absolute level of T-cells was lower in group 2 compared to group 1 (1,002 vs. 1,345; p=0,011). When comparing the relative number of lymphocyte subpopulations, a statistically significant decrease in the content of lymphocytes (15,6 vs. 22,88; p=0,033) and T-cytotoxic cells (20,1 vs. 27,0; p=0,025) in group 3 compared to group 1 was identified. In contrast, an increase in the number of T-helpers, NK-cells and true natural killer cells was observed in group 3, however the differences did not reach the level of statistical significance.

### Identification of Correlations of Lymphocyte Subpopulations With Biochemical Parameters in Blood

**Figure 2:**
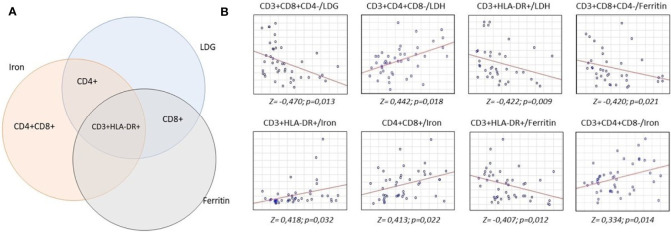
Analysis of correlations of serum concentrations of lactate dehydrogenase, iron and ferritin with lymphocyte subpopulations. 16 ml of whole venous blood out of 43 patients was analyzed to measure lactate dehydrogenase, iron and ferritin levels and lymphocyte subpopulation count. Biochemical analysis was carried out by the colorimetric method, lymphocyte subpopulations studied by flow cytometry. Statistical analysis to find the relationship between these parameters was carried out according to Spearman test. **(A)** intersection of correlations between biochemical parameters and lymphocyte subpopulations. **(B)** revealed positive (upward axis) and negative (downward axis) correlations between lymphocyte subpopulations and biochemical parameters.

After a quantitative study of the main subpopulations of lymphocytes, we carried out a correlation analysis between the main clinical and laboratory parameters and the results of the immunophenotyping. A number of correlations were found between the studied subpopulations, both in absolute and relative terms with the levels of LDH, ferritin and serum iron (**Figure 2**).

The absolute number of T-cytotoxic lymphocytes negatively correlated with the levels of LDH (Z = 0,470) and ferritin (Z = 0,420). We found a positive correlation between the absolute number of T-helpers and serum iron levels (Z = 0,334) and the relative number of T-helpers with LDH levels (Z = 0,442). The absolute number of double positive T-lymphocytes positively correlated with the level of serum iron (Z = 0,413). The greatest number of correlations was found for the absolute number of T-activated lymphocytes - negative with the levels of LDH (Z = 0,422) and ferritin (Z = 0,407) and positive with the levels of serum iron (Z = 0,418).

**Figure 3:**
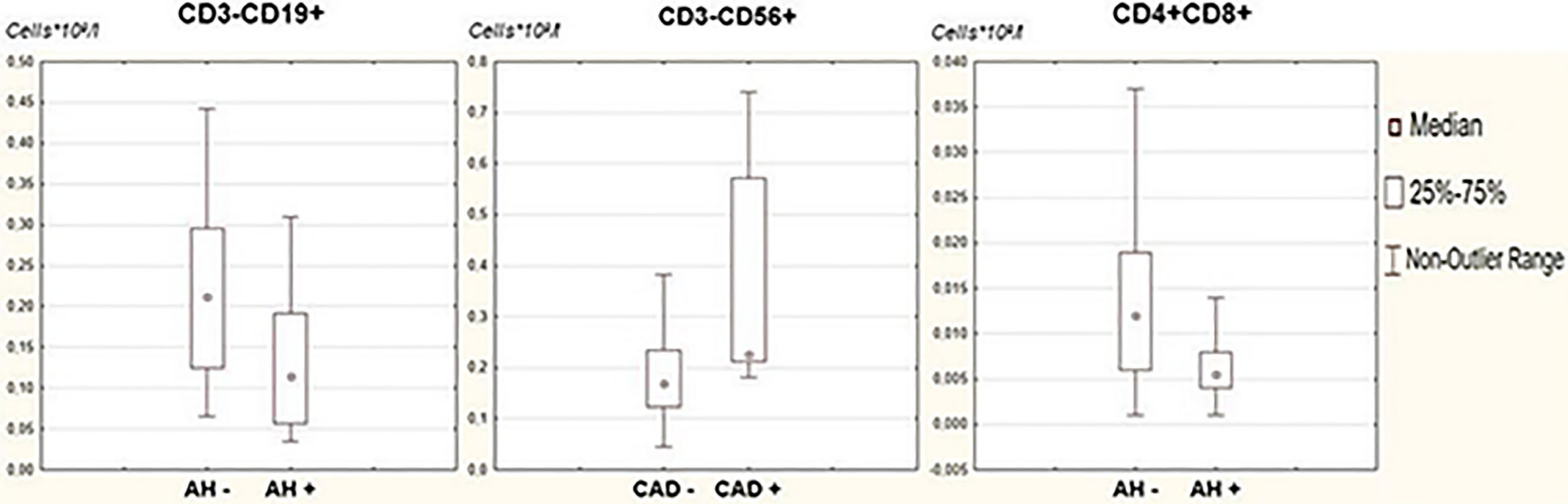
Comparative analysis of lymphocyte subpopulations in patients with associated clinical conditions. Clinical conditions associated with metabolic syndrome - arterial hypertension, overweight, hypercholesterolemia, diabetes mellitus, coronary and cerebral artery disease - were assessed in the study group. For all examined subjects, lymphocyte subpopulations were assessed by flow cytometry. A comparative analysis of lymphocyte subpopulations in patients in the studied groups was carried out; statistical analysis was carried out using the Mann-Whitney test.

When analyzing the distribution of lymphocyte subpopulations, depending on the presence of associated clinical conditions, we found that the absolute numbers of B-lymphocytes and double positive T-lymphocytes in the peripheral blood were statistically significantly reduced in patients with arterial hypertension (p=0,0074 and p=0,0227, correspondingly). This may be due to higher levels of inflammation and suppression of nonspecific immunity. Patients with a history of coronary heart disease have a statistically significantly higher NK cell level (p=0,0108) (**Figure 3**).

## Discussion

Clinical and laboratory markers of the COVID-19 severity are intensively studied by scientists around the world. The basic research, available both in clinics and at the ambulance, is a general blood test, that`s why it can be used as a frequent and usable biomarker. One of the potential markers is lymphopenia, which is mentioned most often (13, 14). Ghahramani et al. (15) in wide a meta-analysis, which included 17 publications, showed a significant decrease in the number of lymphocytes, as well as monocytes and eosinophils, hemoglobin, platelets, an increase in the neutrophils number in patients with severe disease. No significant differences were found in the level of erythrocytes, which is consistent with our results. The underlying cause of lymphopenia in severe cases of COVID-19 is still unknown, and several mechanisms have been proposed to explain it. Some of these hypotheses are apoptosis of T-lymphocytes, IL-induced pyroptosis-1β (16), direct cytopathic effect of the virus on T-lymphocytes (17), bone marrow suppression due to cytokine storm, similar to that in sepsis (18).

The results of biochemical diagnostics in patients with a new coronavirus infection are also under intensive investigation. Jurado et al. found that during hospitalization the levels of interleukin 6, CRP, ferritin, D-dimer, LDH, leukocytes and neutrophils in all patients were higher than the reference values (19). A meta-analysis of data from 1,745 COVID-19 patients from six studies showed that 16% of COVID-19 patients have increased levels of alanine transaminase and aspartate aminotransferase levels in the circulation. 34% of patients had albumin levels below normal, and 6% of patients had elevated total bilirubin levels. Creatinine levels were elevated in 8% of patients, creatine kinase levels were above the normal range in 13% of patients, and 52% of patients had elevated lactate dehydrogenase (LDH) levels, which was recognized as a marker of the severity of the COVID-19 (20). Our study demonstrated, the level of LDH are statistically significantly increased depending on the severity of inflammation, and negatively correlate with the levels of T-helpers and T-cytotoxic lymphocytes. The most diagnostically significant marker of the severity of COVID-19 is C-reactive protein. Tan et al. (21) showed that CRP has good diagnostic accuracy for early prognosis of severe COVID-19 at a cut-off value of 20.42 mg/L. The pooled results for the new combination markers showed a significant increase in the neutrophil-lymphocyte ratio and a decrease in the lymphocyte-C-reactive protein index in hospitalized patients with severe and mild severe cases (22). Overall, the published data suggesting that in the early stages of COVID-19, CRP levels may reflect disease severity, are in line with our findings.

There are several studies that examined the lymphocyte subtypes in patient with COVID-19 where different criteria were chosen to assess the severity of disease. When studying subpopulations of lymphocytes in patients with COVID-19, Odak, I. et al. showed the absolute number of lymphocyte subpopulations was significantly reduced in patients with COVID-19, depending on the severity of clinical symptoms. This is generally consistent with our results, except that oxygen dependence was chosen as a criterion of severity (23). In another study, it was found that the number of CD8+, CD4+, CD19+ and NK cells were reduced in severe cases of the disease. Differences in the CD8 were significant only in patients of 45-60 age group, and natural killer cells in the 60-75 years patients. The decrease in the number of lymphocytes, which was published to date, was found only in the age groups of 30–45 and 45–60 years. In our study, the average age of the participants was 60,0 (50,0-69,0), and significant correlations were identified for the absolute number of lymphocytes, B-cells, T-cells, T-cytotoxic and T-activated cells (24). Rutkowska et al. found a lower proportion of all lymphocytes, T-lymphocytes, B-lymphocytes and increasing of neutrophil counts in patients with severe and extremely severe COVID-19 diagnosed by CT scan results. Decrease of CD8+cells number did not reach the significance, which may be due to the small sample sizes (23 and 12 people, respectively) (25). Moratto, D. et al. presented data with significant differences in the number of CD3+, CD4+ and CD8+lymphocytes among patients with moderate, severe and critical phenotype, the severity condition was assessed with the Sixth Revised Trial Version of the Novel Coronavirus Pneumonia Diagnosis and Treatment Guidance, Italy (26). Liu, Z. et al. found that low CD4+ and CD8+ T lymphocyte counts are more common in patients with severe disease. The lymphocyte subpopulations were also evaluated depending on the presence of comorbidity, the duration of treatment before hospitalization, and the time to RT-PCR turning negative. The significant decrease of the CD8 subpopulation in the group with comorbidity was achieved, while in our report – for double positive T-lymphocytes (CD4+CD8+). There was also a trend to decrease in the number of B-lymphocytes and NK cells in all compared groups, but statistical significance was not achieved due to small samples (39 patients in total) (27). Sun H.B. et al. (28) showed that lymphopenia in patients with COVID-19 is mainly manifested by a decrease in the number of CD4+ T-lymphocytes and correlates with the severity of the disease. The counts and percentages of NK cells, CD4+ T cells, CD8+ T cells and NK-T cells were significantly reduced in patients with severe symptoms, which is consistent with our results. The cytotoxic CD3-CD56^dim^CD16+ cell population significantly decreased, while the CD3-CD56^dim^CD16- part significantly increased in severe COVID-19 patients. We found an increase in the relative number of true natural killers (CD3-CD56+) and T-helpers (CD3+CD4+CD8-) in patients with high CRP levels, but the differences did not reach significance (29). In another study, 112 patients who died from COVID-19 were older and had a higher incidence of comorbidities than those who survived. A significant reduction in total lymphocytes, CD3+, CD4+, CD8+ and CD19+ counts and CD3+ percentage was found in the group of patients (P <0 001), while the percentage of CD56+/CD16+ NK cells was significantly higher (P <0,001). Multivariate logistic regression analysis showed a significantly increased risk of in-hospital death associated to CD4+ T counts ≤ 500 cells/μl, (OR = 2,79, 95% CI = 1,1-6,7; P = 0 021); CD8+ T counts ≤ 100 cells/μl, (OR = 1,98, 95% CI = 1,2-3,3; P = 0,009) and CD56+/CD16+ NK ≥ 30%, (OR = 1,97, 95% CI = 1,1 – 3,1; P = 0,002) at admission, independent of total lymphocyte numbers and co-morbidities. According to our results, the described level of reduction was found for CD4 (0,474 ± 0,198 cell × 10^9^/l) with the highest CRP levels, however, hospitalization for all patients in this group ended with discharge, while study of Cantenys‐Molina et al. describes patients with the lethal outcome (30). It was also reported that changes in lymphocyte subpopulations content remained in patients after the discharge. This study has analyzed 60 patients, and identified that the decrease in the amount of CD8 T cells and B cells, and an increase of CD4/CD8 ratio one-week post-treatment were associated with poor treatment efficacy (31). Decreased absolute counts of CD3+ T cells, CD4+ T cells, CD8+ T cells, and CD45+ T cells were found in both survival group and death group patients during first 3 days of hospitalisation (32). During days 4 to 35 of hospitalisation, that levels of T-cell subsets were gradually normalising in the group of patients who survived, but not in patients with mortal outcome (32). We also plan to harvest a follow-up data for our patients’ cohort. It is of particular importance since the information about the correlations between the lymphocyte subsets and levels of CRP in COVID-19 patients is extremely limited at the moment. Thus, the study by Mangano et al. that enrolled 33 COVID-19 patients revealed negative correlations between the levels of CRP and CD3+, CD3+CD4 +, CD3+ CD8+ subpopulations of lymphocytes, which is consistent with our results (33).

The concentration of LDH, aspartate aminotransferase and sodium ions significantly correlated (P <0.05) with the total lymphocyte count and the count of all lymphocyte subpopulations in patients with COVID-19. Among subpopulations of lymphocytes, the number of CD4+ T cells showed a significant correlation with most biochemical parameters. In contrast, no significant correlation was found between biochemical parameters and the number of monocytes and neutrophils (34). According to our results, the main subpopulations of lymphocytes (CD4+, CD8+, CD4+CD8+) had a negative correlation with the levels of iron, ferritin and LDH in various combinations.

## Conclusion

Thus, this study shows deficiencies in the adaptive immune cells subsets and increased levels of CRP, indicator of systemic inflammation, correlated in patients with COVID.19. The pre-existing cardio-metabolic conditions predisposed the patients to the more severe imbalance on the adaptive and innate immune cell subsets, that can be explained by the systemic disturbances in the immune system preceding onset of COVID-19 disease.

## Data Availability Statement

The original contributions presented in the study are included in the article/**Supplementary Material**. Further inquiries can be directed to the corresponding author.

## Ethics Statement

The studies involving human participants were reviewed and approved by Bashkir State Medical University (protocol №11, 2020). The patients/participants provided their written i nformed consent to participate in this study.

## Author Contributions

AT, VP, and JK designed the project. AT, MS, IM, RK, and AS performed experiments. AT, MS, VP, and JK have analyzed data. AT and JK wrote the manuscript. All authors contributed to the article and approved the submitted version.

## Funding

This study was supported by by a grant from the Republic of Bashkortostan for young scientists SEC-GMU-2021, the Tomsk State University Development Programme (Priority-2030) and Bashkir State Medical University Development Programme (Priority-2030).

## Conflict of Interest

The authors declare that the research was conducted in the absence of any commercial or financial relationships that could be construed as a potential conflict of interest.

## Publisher’s Note

All claims expressed in this article are solely those of the authors and do not necessarily represent those of their affiliated organizations, or those of the publisher, the editors and the reviewers. Any product that may be evaluated in this article, or claim that may be made by its manufacturer, is not guaranteed or endorsed by the publisher.
