# Supplementary material for: Correlation of the Imbalance in the Circulating Lymphocyte Subsets With C-Reactive Protein and Cardio-Metabolic Conditions in Patients With COVID-19
Source: Front Immunol. 2022 May 6;13:856883. doi: 10.3389/fimmu.2022.856883 (PMC9120577; doi:10.3389/fimmu.2022.856883)
Supplement: Supplementary file 1 [file Table_1.docx]

Table. 1. The absolute number of lymphocyte subpopulations in COVID-19 patients grouped according to the CRP levels.

| Subpopulation | C-RP <10  mg/l  Gr. 1 N=17 | C-RP 10-50  mg/l  Gr. 2 N=16 | C-RP >51  mg/l  Gr. 3 N=10 |
| --- | --- | --- | --- |
| Lymphocytes (CD45 bright), cells×10^9^/l, M±SD | 1,541±0,524 | 1,289±0,607 | 1,043±0,459** |
| B-cells (CD3-CD19+), cells×10^9^/l, M±SD | 0,202±0,089 | 0,169±0,118 | 0,112±0,072** |
| T-lymphocytes (CD3+CD19-), cells×10^9^/l, M±SD | 1,345±0,542* | 1,002±0,662 | 0,742±0,278** |
| T-helpers (CD3+CD4+CD8-), cells×10^9^/l, M±SD | 0,658±0,238 | 0,539±0,277 | 0,474±0,198 |
| T-cytotoxic (CD3+CD8+CD4-), cells×10^9^/l, M±SD | 0,440±0,205 | 0,335±0,248 | 0,206±0,102** |
| True natural killers (CD3+CD56+), cells×10^9^/l, M±SD | 0,121±0,082 | 0,075±0,060 | 0,086±0,083 |
| Double positive T-lymphocytes (CD4+CD8+),  cells×10^9^/l, M±SD | 0,015±0,017 | 0,014±0,024 | 0,010±0,012 |
| True natural killers (CD3-CD56+), cells×10^9^/l, M±SD | 0,221±0,119 | 0,214±0,105 | 0,210±0,204 |
| T cells activated (CD3+HLA-DR+), cells×10^9^/l, M±SD | 0,076±0,032 | 0,069±0,055 | 0,041±0,031** |

*Note: * level p <0,5 Gr. 1 vs Gr. 2; ** level p <0,05 Gr. 1 vs Gr. 3; *** level p <0,05 Gr. 2 vs Gr. 3.*
