# Supplementary material for: Correlation of the Imbalance in the Circulating Lymphocyte Subsets With C-Reactive Protein and Cardio-Metabolic Conditions in Patients With COVID-19
Source: Front Immunol. 2022 May 6;13:856883. doi: 10.3389/fimmu.2022.856883 (PMC9120577; doi:10.3389/fimmu.2022.856883)
Supplement: Supplementary file 2 [file Table_2.docx]

Table. 2. The relative number of lymphocyte subpopulations in COVID-19 patients grouped according to the CRP levels.

| Subpopulation | C-RP <10  mg/l  Gr. 1 N=17 | C-RP 10-50  mg/l  Gr. 2 N=16 | C-RP >51  mg/l  Gr. 3 N=10 |
| --- | --- | --- | --- |
| Lymphocytes (CD45 bright), %, M±SD | 22,882±6,882 | 19,413±7,426 | 15,60±3,406** |
| B cells (CD3-CD19+), %, M±SD | 11,647±5,150 | 12,263±6,014 | 10,20±4,530 |
| T-lymphocytes (CD3+CD19-), %, M±SD | 72,706±10,540 | 68,344±9,938 | 69,80±8,954 |
| T-helpers (CD3+CD4+CD8-), %, M±SD | 40,765±9,011 | 41,875±7,779 | 45,50±10,607 |
| T-cytotoxic (CD3+CD8+CD4-), %, M±SD | 27,0±8,796 | 24,938±9,110 | 20,10±6,790** |
| True natural killers (CD3+CD56+), %, M±SD | 6,553±3,452 | 5,563±4,518 | 7,54±5,697 |
| Double positive T-lymphocytes (CD4+CD8+),  %, M±SD | 1,162±0,931 | 1,221±0,943 | 1,228±1,336 |
| True natural killers (CD3-CD56+), %, M±SD | 13,076±7,422 | 16,688±8,725 | 18,0±8,969 |
| T cells activated (CD3+HLA-DR+), %, M±SD | 4,912±2,185 | 4,838±2,502 | 3,86±2,133 |

*Note: * level p <0,05 Gr. 1 vs Gr. 2; ** level p <0,05 Gr. 1 vs Gr. 3; *** level p <0,05 Gr. 2 vs Gr. 3..*
