# Supplementary material for: Correlation of the Imbalance in the Circulating Lymphocyte Subsets With C-Reactive Protein and Cardio-Metabolic Conditions in Patients With COVID-19
Source: Front Immunol. 2022 May 6;13:856883. doi: 10.3389/fimmu.2022.856883 (PMC9120577; doi:10.3389/fimmu.2022.856883)
Supplement: Supplementary file 3 [file Table_3.docx]

Table. 3. Correlations of the main subpopulations of lymphocytes with clinical parameters in COVID-19 patients.

| Subpopulation | Parameter | Correlation level for p<0,05 | |
| --- | --- | --- | --- |
|  |  | Absolute number | Relative number |
| Lymphocytes (CD45 bright) | Iron, μmol / l | 0,310 | - |
|  | Hospitalization, days | -0,401 | - |
| B cells (CD3-CD19+) | Age, years | -0,359 | - |
| T-helpers (CD3+CD4+CD8-) | LDH, Unit | - | 0,442 |
|  | Iron, μmol / l | 0,334 | - |
| T-cytotoxic (CD3+CD8+CD4-) | LDH, Unit | -0,470 | -0,443 |
|  | Ferritin, mcg / l | -0,420 | -0,430 |
|  | Hospitalization, days | -0,350 | - |
| Double positive T-lymphocytes (CD4+CD8+) | Iron, μmol / l | 0,413 | -0,440 |
|  | Age, years | -0,410 | -0,35 |
| T cells activated (CD3+HLA-DR+) | LDH, Unit | -0,422 | -0,340 |
|  | Iron, μmol / l | 0,418 | - |
|  | Ferritin, mcg / l | -0,407 | -0,400 |
|  | Before hospitalization, days | -0,355 | - |
