# Supplementary material for: Correlation of the Imbalance in the Circulating Lymphocyte Subsets With C-Reactive Protein and Cardio-Metabolic Conditions in Patients With COVID-19
Source: Front Immunol. 2022 May 6;13:856883. doi: 10.3389/fimmu.2022.856883 (PMC9120577; doi:10.3389/fimmu.2022.856883)
Supplement: Supplementary file 4 [file Table_4.docx]

Tab. 4. The absolute number of the main subpopulations of lymphocytes in patients with associated clinical conditions

| Variable | Diabetes | | Arterial  Hypertension | | Hypercoles- terolemia | | Coronary Artery Disease | | Cerebro­vascular Disease | |
| --- | --- | --- | --- | --- | --- | --- | --- | --- | --- | --- |
|  | +  N=6 | -  N=37 | +  N=22 | -  N=21 | +  N=3 | -  N=40 | +  N=7 | -  N=36 | +  N=13 | -  N=30 |
| CD45+, cells×10^9^/l, M±SD | 1,24±0,38 | 1,34±0,59 | 1,26±0,56 | 1,4±0,57 | 1,34±0,38 | 1,34±0,54 | 1,15±0,38 | 1,29±0,59 | 1,43±0,7 | 1,28±0,5 |
| P value | 0,7658 | | 0,3075 | | 0,9810 | | 0,2363 | | 0,6153 | |
| CD3-CD19+, cells×10^9^/l, M±SD | 0,18±0,06 | 0,14  [0,071; 0,25]* | 0,13±0,07 | 0,21±0,1 | 0,2±0,1 | 0,16±0,1 | 0,19±0,11 | 0,16±0,09 | 0,16±0,1 | 0,17±0,1 |
| P value | 0,4952 | | 0,0074 | | 0,4721 | | 0,5216 | | 0,9770 | |
| CD3+CD19-, cells×10^9^/l, M±SD | 0,88±0,25 | 0,87  [0,71; 1,37]* | 1,16±0,56 | 1,16  [0,79; 1,18]* | 1,23±0,33 | 0,89  [0,7; 1,16]* | 1,1±0,31 | 0,86  [0,7; 1,24]* | 1,23±0,77 | 0,89  [0,71; 1,15]* |
| P value | 0,7391 | | 0,2244 | | 0,3284 | | 0,2704 | | 0,4510 | |
| CD3+CD4+CD8-, cells×10^9^/l, M±SD | 0,56±0,19 | 0,51  [0,35; 0,67]* | 0,59±0,22 | 0,55±0,27 | 0,68±0,18 | 0,56±0,25 | 0,63±0,19 | 0,55±0,26 | 0,63±0,35 | 0,54±0,19 |
| P value | 0,7926 | | 0,4019 | | 0,2834 | | 0,2298 | | 0,6435 | |
| CD3+CD8+CD4-, cells×10^9^/l, M±SD | 0,26±0,15 | 0,29  [0,21; 0,44]* | 0,25  [0,17; 0,42]* | 0,38  [0,24; 0,55]* | 0,32±0,1 | 0,26  [0,18; 0,45]* | 0,29±0,11 | 0,27  [0,18; 0,46]* | 0,39±0,27 | 0,25  [0,21; 0,43]* |
| P value | 0,3264 | | 0,1661 | | 0,9051 | | 0,8953 | | 0,5877 | |
| CD3+CD56+, cells×10^9^/l, M±SD | 0,071±0,05 | 0,06  [0,04; 0,15]* | 0,09±0,07 | 0,09  [0,04; 0,13]* | 0,15±0,11 | 0,06  [0,03; 0,14]* | 0,12±0,09 | 0,06  [0,03; 0,14]* | 0,1±0,08 | 0,06  [0,03; 0,13]* |
| P value | 0,7524 | | 0,8841 | | 0,2525 | | 0,4297 | | 0,5877 | |
| CD4+CD8+, cells×10^9^/l, M±SD | 0,0068  [0,005; 0,007]* | 0,008  [0,005; 0,15]* | 0,005  [0,004; 0,008]* | 0,016  [0,006; 0,19]* | 0,01±0,003 | 0,006  [0,005; 0,14]* | 0,008±0,005 | 0,007  [0,005; 0,14]* | 0,006  [0,005; 0,12]* | 0,007  [0,005; 0,15]* |
| P value | 0,5154 | | 0,0227 | | 0,3763 | | 0,5636 | | 0,5595 | |
| CD3-CD56+, cells×10^9^/l, M±SD | 0,19±0,10 | 0,18  [0,13; 0,25]* | 0,18  [0,13; 0,33]* | 0,2  [0,14; 0,22]* | 0,18±0,04 | 0,18  [0,13; 0,29]* | 0,22  [0,21; 0,57]* | 0,16  [0,12; 0,23]* | 0,18±0,09 | 0,19  [0,13; 0,3]* |
| P value | 0,6613 | | 0,7986 | | 0,9810 | | 0,0108 | | 0,6061 | |
| CD3+HLADR, cells×10^9^/l, M±SD | 0,059  [0,024; 0,08]* | 0,06  [0,03; 0,09]* | 0,07±0,04 | 0,06±0,04 | 0,08±0,012 | 0,06  [0,03; 0,09]* | 0,06±0,03 | 0,05  [0,03; 0,09]* | 0,07±0,05 | 0,06±0,03 |
| P value | 0,4615 | | 0,1481 | | 0,3052 | | 0,6450 | | 0,7711 | |

*Note: * - Me [Q1; Q3] range.*
